# Factors associated with Saudi nurses’ knowledge, attitudes, and practices in preventing catheter-associated urinary tract infection in Aljouf City: a cross-sectional study

**DOI:** 10.3389/fpubh.2026.1781575

**Published:** 2026-04-02

**Authors:** Bahia Galal Abd Elrazik Siam, Khalda Mnawer M. Alshammari, Bassam Shayhan F. Alshammari, Aiman Mojidea M. Alshammari, Saad Majed Saad Alderaan, Saad Thamer Alshammari, Abdulellah Nasser Abdulaziz Alshammari, Omar Nasser Saud Almaslamani, Salman Amish Ayad Alshammari, Wadha Habeeb Almatrafy, Faisal Dakhilallah J. Alruwaili, Abdulaziz Fayez L. Alruwaili, Jawaher Jaza M. Alruwaili, Mashael Musallam Alahmadi, Mona Lahilam Alsamah Aldoghmani, Wejdan Sager S. Alrwaili, Mashal Khaled Alrowily, Hamdah Falgi Basheer Alruwaili

**Affiliations:** 1 College of Nursing, University of Hail, Hail, Saudi Arabia; 2 Al-Jouf Health Cluster, Ministry of Health, Sakaka, Saudi Arabia; 3 Prince Muteb Bin Abdulaziz Hospital, Al-Jouf Health Cluster, Ministry of Health, Sakaka, Saudi Arabia; 4 King Khaled Hospital, Ha’il Cluster, Hail, Saudi Arabia; 5 Nursing Executive Administration, Hail Health Cluster, Hail, Saudi Arabia; 6 Suwair General Hospital, Al-Jouf Health Cluster, Sakaka, Saudi Arabia; 7 King Salman Bin Abdulaziz Medical City, Madinah Health Cluster, Madinah, Saudi Arabia

**Keywords:** attitudes, catheter-associated urinary tract infection, CAUTI, factors associated, knowledge, nurses, practices, Saudi Arabia

## Abstract

**Background:**

Catheter-associated urinary tract infection (CAUTI) is a leading healthcare-associated infection worldwide, associated with increased morbidity, prolonged hospitalization, higher costs, and antimicrobial resistance. Nurses play a critical role in prevention through evidence-based catheter insertion, maintenance, and timely removal.

**Aim:**

To assess factors associated with knowledge, attitudes, and preventive practices regarding CAUTI among nurses in Aljouf City, Saudi Arabia.

**Methods:**

A descriptive cross-sectional study was conducted among 184 registered nurses in governmental hospitals in Aljouf City using a structured questionnaire on demographics, knowledge, attitudes, and practices (KAP) toward CAUTI prevention. Data were analyzed with descriptive statistics, independent t-tests, ANOVA, and Spearman’s correlations.

**Results:**

Overall, 77.7% of nurses had good knowledge, 84.8% showed positive attitudes, and 84.8% had adequate preventive practices. Knowledge scores differed significantly by years of experience (*p* = 0.001), urinary catheter (UC) insertion frequency (*p* < 0.001), and CAUTI guideline awareness (*p* = 0.002). Attitudes varied by hospital (*p* = 0.014), ward (*p* = 0.007), and UC insertion frequency (*p* = 0.032). Practices differed by hospital (*p* = 0.006), UC insertion frequency (*p* = 0.009), and guideline awareness (*p* = 0.049). Spearman’s correlations showed weak knowledge-attitude (*r* = 0.231, *p* = 0.002), moderate knowledge-practice (*r* = 0.446, *p* < 0.001), and strong attitude-practice (*r* = 0.525, p < 0.001) associations. Multivariable logistic regression showed that attitude was the strongest independent factor associated with adequate practice (AOR = 0.251, *p* = 0.005).

**Conclusion:**

This study demonstrates generally favorable knowledge, attitudes, and practices toward CAUTI prevention among Al-Jouf nurses, with moderate correlations, attitudes showed the strongest association with preventive practices.

## Introduction

Catheter-associated urinary tract infection (CAUTI) is among the most common and preventable healthcare-associated infections (HAIs), linked to increased morbidity, prolonged hospitalization, higher costs, and antimicrobial resistance (1). While urinary catheterization is often essential, infection risk rises with non-aseptic insertion, prolonged dwell time, or inadequate maintenance (2–4), leading to avoidable patient discomfort, extended hospital stays, and even mortality (5). These factors highlight the need for strict adherence to evidence-based preventive measures and continuous evaluation of clinical practice.

Nurses play a central role in CAUTI prevention, as they are primarily responsible for catheter insertion, maintenance, daily monitoring, and timely removal. Evidence-based guidelines emphasize several essential preventive measures, including aseptic insertion using sterile equipment, maintaining a closed drainage system, selecting the appropriate catheter size, performing regular perineal hygiene, and conducting daily assessments of catheter necessity (1, 6). However, consistent adherence to these practices depends largely on nurses’ knowledge, attitudes, and clinical behaviors (7).

Knowledge, attitude, and preventive practice (KAP) are interrelated factors influencing nurses’ effectiveness in CAUTI prevention. Adequate knowledge about catheter indications, aseptic techniques, and maintenance protocols provides the foundation for safe clinical practice. Positive attitudes toward infection prevention, including recognizing the importance of adherence to guidelines and prioritizing patient safety, influence motivation and consistency in applying preventive measures. However, evidence consistently documents knowledge-practice gaps in CAUTI prevention, where nurses demonstrate adequate knowledge but suboptimal bedside adherence (8, 9).

Despite availability of evidence-based guidelines, studies consistently report gaps in nurses’ CAUTI knowledge and variable protocol adherence. Barriers including high workload, time constraints, limited training, and inconsistent organizational support hinder implementation (10, 11). International evidence further emphasizes persistent protocol non-adherence, highlighting the need for standardized care bundles, continuous auditing, and targeted training interventions (12, 13).

In Saudi Arabia, reducing HAIs is a central component of the national health transformation under Vision 2030. While national infection prevention guidelines emphasize CAUTI reduction, healthcare institutions continue to face challenges such as staffing shortages, variable training quality, and inconsistent policy implementation (14). Regional studies document variations in nurses’ CAUTI knowledge and preventive practices across hospitals, highlighting needs for localized assessments to guide targeted interventions (15).

Despite the importance of CAUTI prevention, limited research has explored nurses’ KAP in northern Saudi Arabia, particularly Al-Jouf City. Addressing this regional gap is essential for identifying performance deficits, understanding contextual barriers, and informing targeted training and policy initiatives. Therefore, this study aimed to assess factors associated with nurses’ knowledge, attitudes, and preventive practices regarding CAUTI in Aljouf City, Saudi Arabia.

## Methods

### Study design

A descriptive cross-sectional analytical study was conducted to evaluate nurses’ knowledge, attitudes, and preventive practices related to catheter-associated urinary tract infection (CAUTI) prevention. This design allowed assessment of associations but not causal relationships.

### Study setting

The study was carried out in four governmental hospitals in Aljouf City, Saudi Arabia: Domat Al-Jandal Hospital (Doma. H), King Abdulaziz Specialist Hospital (KASH), Maternity and Children Hospital (MCH), and Prince Muteb Hospital (PMH). These hospitals include general wards and intensive care units (ICUs) where urinary catheterization is routinely performed. The diversity of clinical units supports broader representativeness within the region.

### Target population

Registered nurses providing direct patient care in units involving urinary catheter insertion, maintenance, or removal in governmental hospitals in Aljouf City, Saudi Arabia.

#### Inclusion criteria

- Registered nurses employed in Aljouf governmental hospitals
- At least 6 months of clinical experience
- Direct involvement in urinary catheter care
- Willing and able to provide informed consent

#### Exclusion criteria

- Nursing students or interns
- Nurses in administrative or non-clinical roles

### Sample size and sampling technique

Using Cochran’s formula at 95% confidence level, 5% margin of error, and 50% anticipated prevalence, the minimum sample size was 162. After accounting for a 10–15% non-response rate, a final sample of 184 nurses was obtained. Stratified random sampling was used, with hospitals as strata. Nurses were then selected from each hospital and unit to reflect ward distribution and ensure adequate representation.

### Data collection tool

Data was collected using a structured, self-administered questionnaire adapted from previous literature (16). The tool comprised four sections:

- Section 1: Demographics and professional characteristics – Age, gender, education level, years of experience, hospital, current ward, and frequency of urinary catheter (UC) insertion in the past month.
- Section 2: Knowledge – 27 closed-ended items on CAUTI indications and prevention methods, scored 1 for correct and 0 for incorrect answers (total range: 0–27). Scores were categorized as poor (<70%, <19) or good (≥19).
- Section 3: Attitudes – 18 items assessing attitudes toward CAUTI prevention, scored 1 for positive and 0 for negative responses (total range: 0–18). Scores were categorized as negative (<70%, <13) or positive (≥13).
- Section 4: Preventive practices – 23 items on self-reported CAUTI prevention practices, scored 1 for adequate and 0 for inadequate responses (total range: 0–23). Scores were categorized as inadequate (<70%, <17) or adequate (≥17).

Higher scores reflected better knowledge, more positive attitudes, and more adequate preventive practices. Content and face validity were established by a panel of five infection control experts. The questionnaire was pilot tested with 15 nurses from a non-participating hospital, yielding good acceptability. Internal consistency was excellent: overall KAP *α* = 0.806; knowledge subscale *α* = 0.82 (27 items); attitudes *α* = 0.78 (18 items); practices *α* = 0.85 (23 items); all >0.70 acceptable.

### Data collection procedure

Data was collected from May to July 2025. Questionnaires were distributed during nursing shifts in coordination with unit managers. Participation was voluntary and anonymous, with informed consent obtained. Completed forms were reviewed immediately for completeness, and non-respondents received one reminder not multiple. The questionnaire was developed in English and translated into Arabic using forward-backward translation to ensure linguistic and semantic equivalence, verified by bilingual experts.

### Data analysis

Data analysis was performed using IBM SPSS Statistics version 27. Descriptive statistics summarize categorical variables as frequencies and percentages, and continuous variables as means ± standard deviations. Normality was assessed using Shapiro–Wilk tests (all *p* < 0.05, non-normal). Group comparisons used independent t-tests and one-way ANOVA with Games-Howell post-hoc tests (unequal variances). Spearman rho correlations examined KAP associations due to non-normality. Multivariable logistic regression (forward stepwise LR) identified factors independently associated with adequate practice (dependent variable: adequate = 1, inadequate = 0). Model fit assessed via Hosmer-Lemeshow test and Nagelkerke *R*^2^. Missing data (<5%) handled via pairwise deletion. Statistical significance set at *p* < 0.05. This study followed STROBE guidelines for cross-sectional reporting.

### Ethical considerations

Ethical approval was obtained from the University of Hail Research Ethics Committee (Reference No. H-2025-604, dated 10/2/2025). This study involved human participants and complied with national and institutional ethical standards. Written informed consent was secured from all participants via an initial consent question following explanation of study aims, procedures, risks, benefits, and voluntary nature. Anonymity and confidentiality were maintained through coded data storage; participants could withdraw without consequence at any time. Data are stored in password-protected files accessible only to the research team and will be retained for 5 years before secure destruction.

## Results

Table 1 indicates that 63.0% of participant nurses were under 30 years old and 69.0% were female. Most held bachelor’s degrees (83.7%), had 2–6 years of experience (40.8%), and worked in intensive care units (51.6%). Notably, 41.3% inserted urinary catheters 2–3 times in the past month, yet 93.5% lacked CAUTI-specific training and 88.6% were aware of prevention guidelines.

**Table 1:** Sociodemographic and work-related characteristics of participant nurses (*n* = 184).

Variable	Count	(%)
Age (years old)		
<30	116	(63.0)
≥30	68	(37.0)
Gender		
Male	57	(31.0)
Female	127	(69.0)
Education level		
Diploma	21	(11.4)
Bachelor	154	(83.7)
Master	9	(4.9)
Workplace (Hospital)		
Doma. H	33	(17.9)
KASH	47	(25.5)
MCH	42	(22.8)
PMH	62	(33.7)
Current ward		
Intensive care unit	95	(51.6)
General ward	74	(40.2)
Other wards	15	(8.2)
Years of experience		
<2	31	(16.8)
2–6	75	(40.8)
7–10	50	(27.2)
>10	28	(15.2)
Frequency of UC insertion in the past month		
Less than 2 times	41	(22.3)
2–3	76	(41.3)
> 3	67	(36.4)
Received CAUTI education/training		
Yes	12	(6.5)
No	172	(93.5)
Awareness of CAUTI prevention guidelines		
Yes	163	(88.6)
No	21	(11.4)

Table 2 shows that 77.7% of nurses demonstrated good knowledge, 84.8% showed positive attitude, and reported adequate overall practice toward CAUTIs prevention.

**Table 2:** Total scores of nurses’ knowledge, attitudes, and perceived preventive practice toward CAUTI prevention (*n* = 184).

CAUTI prevention variables	Count	(%)	Min	Max	Mean ± SD
Knowledge					
Poor	41	(22.3)			
Good	143	(77.7)	14	27	21.45 **±** 3.30
Attitude					
Negative	28	(15.2)			
Positive	156	(84.8)	7	17	14.71 **±** 2.14
Perceived preventive practice					
Inadequate practice	28	(15.2)			
Adequate practice	156	(84.8)	7	23	18.66 **±** 2.38

Table 3 shows that Post-hoc Games-Howell tests revealed specific group differences: knowledge scores highest among nurses with <2 years of experience vs. 7–10 years (MD = 2.75, *p* = 0.002); attitudes most positive in MCH vs. KASH (MD = 1.46, *p* = 0.013); practices superior in MCH/PMH vs. Domat. H (*p* ≤ 0.006).

**Table 3:** Associations between sociodemographic/occupational variables and nurses’ knowledge, attitudes, and practices (KAP) toward CAUTI prevention (*N* = 184).

Variables	*N*	Knowledge		Attitude		Practice	
		Mean	*p* value	Mean	*p* value	Mean	*p* value
Age (year) ^T^							
≤ 30	116	21.74	0.117	14.7672	0.587	18.7931	0.281
> 30	68	20.96		14.6029		18.4265	
Gender ^T^							
Male	57	22.16	0.056	14.5965	0.641	19.0702	0.116
Female	127	21.14		14.7559		18.4724	
Education level ^F^							
Diploma	21	21.423	0.590	14.9524	0.847	19.0476	0.685
Bachelor	154	21.39		14.6688		18.6234	
Master	9	22.56		14.7778		18.3333	
Workplace (Hospital) ^F^							
Doma. H	33	20.79	0.133	14.7273	**0.014** ^*^	17.5758	**0.006** ^**^
KASH	47	21.28		14.0638		18.3404	
MCH	42	21.03		15.5238		19.2143	
PMH	62	22.23		14.6290		19.0968	
Years of Experience ^F^							
< 2	31	22.65	**0.001** ^*^	13.7742	**0.020** ^*^	18.1290	0.108
2–6	75	21.71		15.1733		18.9467	
7–10	50	19.90		14.5800		18.2200	
> 10	28	22.21		14.7143		19.2500	
Current ward ^F^							
ICU	95	21.93	0.128	14.7158	**0.007** ^**^	18.6421	0.939
General	74	20.97		15.0135		18.6351	
Other	15	20.80		13.1333		18.8667	
UC insertion in the past month ^F^							
0–1	41	22.34	**0.000** ^**^	13.9756	**0.032** ^*^	17.8780	**0.009** ^**^
2–3	76	20.18		15.0526		18.5263	
> 3	67	22.34		14.7612		19.2836	
Received CAUTI Education/Training ^T^							
Yes	12	22.33	0.339	14.17	0.544	19.00	0.677
No	172	21.39		14.74		18.64	
Awareness of CAUTI prevention guidelines ^T^							
Yes	163	23.53	**0.002** ^**^	14.72	0.843	19.62	**0.049** ^*^
No	21	21.18		14.62		18.54	

Bold values indicate statistically significant results (*p* < 0.05). *F-tests followed by Games-Howell post-hoc (unequal variances): Knowledge by experience - < 2 vs 7–10 years (*p* = 0.002, MD = 2.75); Attitudes by hospital–MCH vs KASH (*p* = 0.013); Practices by hospital - MCH vs Domat. H (*p* = 0.005). All adjusted for multiple comparisons.

Table 4. Spearman correlations confirmed moderate knowledge-practice (*ρ* = 0.45, *p* < 0.001) and attitude-practice (*ρ* = 0.53, *p* < 0.001) associations.

**Table 4:** Spearman correlations between nurses’ knowledge, attitudes, and practices toward CAUTI prevention.

Variables	Correlation	Coefficient	Knowledge	Practice	Attitude
Spearman’s rho	Knowledge	Correlation Coefficient	1.000	0.446^**^	0.231^**^
		Sig. (2-tailed)	–	0.000	0.002
		*N*	184	184	184
	Practice	Correlation Coefficient	0.446^**^	1.000	0.525^**^
		Sig. (2-tailed)	0.000	–	0.000
		*N*	184	184	184
	Attitude	Correlation Coefficient	0.231^**^	0.525^**^	1.000
		Sig. (2-tailed)	0.002	0.000	–
		*N*	184	184	184

**Correlation is significant at the 0.01 level (2-tailed).

**Figure 1:**
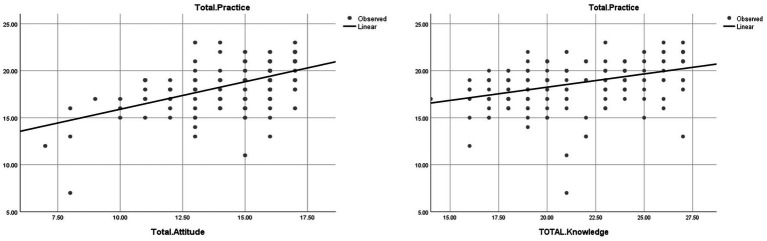
Scatter plots of nurses’ knowledge, attitudes, and practices toward CAUTI prevention (*N* = 184).

Figure 1 illustrates moderate positive correlations: knowledge vs. practice (Spearman’s *r* = 0.446, *p* < 0.001) and attitudes vs. practice (*r* = 0.525, *p* < 0.001), with upward trends confirming statistical associations.

Table 5. Multivariable logistic regression (forward LR) showed negative attitude independently associated with inadequate practice (AOR = 0.25, 95%CI: 0.10–0.66, *p* = 0.005), adjusting for knowledge (AOR = 0.44, 95%CI: 0.17–1.11, *p* = 0.081). Model demonstrated acceptable fit (Hosmer-Lemeshow *p* = 0.41).

**Table 5:** Multivariable logistic regression: factors associated with adequate CAUTI preventive practices (*n* = 184).

Variable	B	SE	Wald	*p*-value	AOR	95% CI for AOR
Knowledge						
Poor knowledgeᵃ	−0.827	0.473	3.053	0.081	0.438	0.173–1.106
Attitude						
Negative attitudeᵇ	−1.382	0.491	7.940	0.005	0.251	0.096–0.657
Constant	2.227	0.302	54.362	<0.001	9.273	—

Hosmer-Lemeshow *χ*^2^ = 8.2, *p* = 0.41; Nagelkerke *R*^2^ = 0.22; −2LL = 156.3. Variable coding: Practice (1 = adequate ≥17, 0 = inadequate <17); Knowledge (1 = poor ≤19, 0 = good >19); Attitude (1 = negative ≤13, 0 = positive >13).

## Discussion

This study assessed knowledge, attitudes, and preventive practices regarding CAUTI among nurses in Al-Jouf City, Saudi Arabia. The predominance of young (<30 years) and female nurses mirrors national and international nursing workforce trends (17, 18). Globally, women comprise 80–90% of nurses, with 25–34-year-olds prominent in low/middle-income countries (19), unlike aging workforces (median mid-40s) in high-income settings like the US (20, 21). This demographic profile suggests that many nurses in the region are early in their careers and may benefit from sustained mentorship and structured in-service training programs.

The high proportion of bachelor’s degree-prepared nurses (83.7%) aligns with recent Saudi findings, where 66% held bachelor’s qualifications (22). Baccalaureate preparation has become standard for hospital nursing, especially in acute care (19, 20). Over half worked in ICUs (51.6%), comparable to CAUTI studies in high-acuity settings where ICU nurses predominate among catheter handlers (23). ICUs typically have higher catheter utilization rates and longer dwell times, underscoring the particular importance of targeted CAUTI training in these units.

Although most nurses reported awareness of CAUTI prevention guidelines (88.6%), only 6.5% received formal training, a pronounced training gap that mirrors findings from other resource-limited or understaffed clinical settings. This aligns with recent CAUTI studies where most nurses reported guideline familiarity, despite variable practice adherence (24, 25). In contrast, the markedly lower proportion of nurses who attended formal CAUTI prevention training in the study by Acharya et al. (26) suggests contextual and institutional variability in educational opportunities.

Concerning nurses’ knowledge of CAUTI prevention (Table 2), over three-quarters demonstrated good knowledge, aligning with recent regional studies reporting majority adequate knowledge (16, 18, 27, 28). However, the findings contrast with others showing knowledge deficits: 62.8% low in Saudi Arabia (17), 42.7% good in Ethiopia (29), 63.0% poor (30), moderate levels (23, 31), and gaps in catheter securing among Spanish ICU nurses (32, 33).

Knowledge scores significantly varied by years of experience (<2 years highest: *M* = 22.65, *p* = 0.001), suggesting that recent graduates may retain more up-to-date theoretical content due to recent education and competency-based curricula. However. Frequency of urinary catheter insertion also significantly associated with nurses’ knowledge (*p* < 0.001), this pattern highlights the need for ongoing training to prevent knowledge decay among mid-career nurses. Less experienced nurses demonstrated superior knowledge, contrary to typical patterns where tenure predicts competence (25, 29, 34), but likely due to recent training aligning with studies where education overrides experience (16). Rashmi and Dhakal (35) similarly found prior CAUTI information significantly predicted knowledge across demographics. These patterns underscore needs for targeted onboarding for newer nurses and continuous education per WHO guidelines (13).

Most nurses (84.8%) demonstrated positive attitudes toward CAUTI prevention (Table 2), aligning with international studies reporting favorable attitudes among trained nursing staff. Mong et al. (16) found positive attitudes in Malaysian hospitals, while Polat et al. (36) reported high scores among Turkish nurses with infection-control education. In contrast, Ethiopian studies document suboptimal attitudes in low-resource settings (29), suggesting Al-Jouf’s profile benefits from relatively stronger institutional supports.

Nurses’ attitudes varied significantly by experience (<2 years lowest: M = 13.77, *p* = 0.020*) and ward (*p* = 0.007**; Table 3). This may reflect unit-level cultures, leadership engagement, or differences in workflow standardization. Novice nurses exhibited less favorable attitudes than mid-career peers (2–6 years: M = 15.17), aligning with studies linking early-career stages to lower motivation before training solidifies EBP commitment (29). Targeted interventions for early-career nurses’ orientation programs, guideline familiarization can boost attitudes, preventing suboptimal patterns from becoming entrenched (13, 37, 38).

Adequate CAUTI prevention practices were reported by 84.8% of nurses (Table 2), aligning with studies documenting good catheter care practices among most nurses (28, 39). In contrast, other research reveals poor practice levels among the majority (17, 40). These discrepancies may reflect differences in institutional policies, infection control oversight, and resource availability across settings.

Practice scores varied significantly by hospital, insertion frequency, and guideline awareness. Nurses inserting catheters more frequently (>3 times/month) and those in hospitals with stronger infection control systems (MCH, PMH) reported higher practice scores. This pattern is consistent with evidence that greater procedural exposure and institutional support can reinforce adherence to CAUTI protocols (38, 41).

Spearman correlation analysis revealed weak positive associations between knowledge and attitudes (*ρ* = 0.231, *p* = 0.002), moderate positive associations between knowledge and practices (*ρ* = 0.446, *p* < 0.001), and strong positive associations between attitudes and practices (*ρ* = 0.525, *p* < 0.001). These align with prior CAUTI KAP studies documenting moderate knowledge-practice correlations (33, 42). Attitudes showed strongest practice association, attitudes are often stronger predictors of preventive behaviors than knowledge alone (16, 29). This underscores attitude-targeted interventions for sustained CAUTI prevention. This suggests that enhancing nurses’ motivation, perceived responsibility, and safety culture may yield greater improvements in CAUTI practices than knowledge-based interventions alone.

The multivariable logistic regression analysis demonstrated that attitude was the only independent factor associated with adequate CAUTI preventive practice after adjusting for knowledge. This finding reinforces the bivariate results showing a stronger attitude–practice correlation than knowledge–practice correlation. In contrast, the association between knowledge and practice did not remain significant in the adjusted model, suggesting that although knowledge contributes to performance, it may not independently predict preventive behavior when attitudinal factors are accounted for. This pattern aligns with previous reports identifying motivational and behavioral determinants as key influences on infection-prevention practices.

### Implications for practice

- Structured and recurring CAUTI-focused training programs are needed, particularly for early-career nurses who demonstrated lower attitude scores.
- Institutional support through audits, feedback, supervision, and bundled protocols can strengthen practice adherence.
- Greater emphasis should be placed on attitude-building strategies, such as reinforcing safety culture, improving interprofessional communication, and engaging frontline staff in policy development.
- Ward-level differences should be addressed through targeted interventions, particularly in units with lower practice scores.

These recommendations align with recent WHO and CDC guidelines advocating multimodal infection control strategies.

### Strengths and limitations

Study strengths include its multicenter design, stratified sampling, validated measurement tool, and satisfactory internal reliability. However, several limitations must be acknowledged:

- The cross-sectional design limits causal inference.
- Self-reported practices may be affected by social desirability bias and may overestimate actual compliance.
- The sample was limited to governmental hospitals in one city, which may limit generalizability.
- The very low proportion of nurses receiving CAUTI-specific training may limit the ability to detect training effects.
- Potential confounders such as staffing ratios and institutional policies were not measured.

Future research should consider longitudinal designs, observational assessments of practice, and multi-regional comparisons.

## Conclusion

In conclusion, nurses in Aljouf City, Saudi Arabia demonstrated favorable levels of knowledge, positive attitudes, and adequate preventive practices, although notable variability was observed across demographic and professional subgroups. Attitudes showed the strongest association with preventive practices, while knowledge demonstrated a moderate association. Significant differences in KAP scores by hospital, experience level, and catheter insertion frequency highlight the need for targeted, context-specific interventions. The particularly low rate of CAUTI-specific training among participants underscores the importance of implementing routine, structured educational programs that reinforce both knowledge and positive safety attitudes. The multivariable analysis confirmed that attitude was the strongest independent factor associated with adequate CAUTI preventive practices, while knowledge was not independently associated after adjustment. These findings highlight the critical role of reinforcing positive attitudes and safety culture to improve preventive behaviors.

## Data Availability

The raw data supporting the conclusions of this article will be made available by the authors, without undue reservation.
